# Correcting bias in cardiac geometries derived from multimodal images using spatiotemporal mapping

**DOI:** 10.1038/s41598-023-33968-5

**Published:** 2023-05-19

**Authors:** Debbie Zhao, Charlène A. Mauger, Kathleen Gilbert, Vicky Y. Wang, Gina M. Quill, Timothy M. Sutton, Boris S. Lowe, Malcolm E. Legget, Peter N. Ruygrok, Robert N. Doughty, João Pedrosa, Jan D’hooge, Alistair A. Young, Martyn P. Nash

**Affiliations:** 1 Auckland Bioengineering Institute, University of Auckland, 70 Symonds Street, Grafton, Auckland, 1010 New Zealand; 2 Department of Anatomy and Medical Imaging, University of Auckland, Auckland, New Zealand; 3 Counties Manukau Health Cardiology, Middlemore Hospital, Auckland, New Zealand; 4 Green Lane Cardiovascular Service, Auckland City Hospital, Auckland, New Zealand; 5 Department of Medicine, University of Auckland, Auckland, New Zealand; 6 Institute for Systems and Computer Engineering, Technology and Science (INESC TEC), Porto, Portugal; 7 Department of Cardiovascular Sciences, KU Leuven, Leuven, Belgium; 8 Department of Biomedical Engineering, King’s College London, London, UK; 9 Department of Engineering Science, University of Auckland, Auckland, New Zealand

**Keywords:** Cardiology, Mathematics and computing, Biomedical engineering

## Abstract

Cardiovascular imaging studies provide a multitude of structural and functional data to better understand disease mechanisms. While pooling data across studies enables more powerful and broader applications, performing quantitative comparisons across datasets with varying acquisition or analysis methods is problematic due to inherent measurement biases specific to each protocol. We show how dynamic time warping and partial least squares regression can be applied to effectively map between left ventricular geometries derived from different imaging modalities and analysis protocols to account for such differences. To demonstrate this method, paired real-time 3D echocardiography (3DE) and cardiac magnetic resonance (CMR) sequences from 138 subjects were used to construct a mapping function between the two modalities to correct for biases in left ventricular clinical cardiac indices, as well as regional shape. Leave-one-out cross-validation revealed a significant reduction in mean bias, narrower limits of agreement, and higher intraclass correlation coefficients for all functional indices between CMR and 3DE geometries after spatiotemporal mapping. Meanwhile, average root mean squared errors between surface coordinates of 3DE and CMR geometries across the cardiac cycle decreased from 7 ± 1 to 4 ± 1 mm for the total study population. Our generalised method for mapping between time-varying cardiac geometries obtained using different acquisition and analysis protocols enables the pooling of data between modalities and the potential for smaller studies to leverage large population databases for quantitative comparisons.

## Introduction

Functional cardiac indices, such as chamber volume fluctuations and strain, can provide important information for the characterisation of pathophysiological mechanisms in cardiac disease. These indices are typically derived from representations of cardiac geometry, obtained from the analysis of images acquired from non-invasive modalities such as 2D or 3D echocardiography (3DE), or cardiac magnetic resonance (CMR) imaging. Technical advancements in imaging have enabled the development of more complex analyses such as cardiac biomechanics for precision medicine^1–4^, and statistical shape modelling^5,6^, which rely on the extraction of accurate cardiac geometries. Furthermore, compared with the analysis of static geometries alone, time-varying geometric models can provide more comprehensive information on changes in cardiac structure and function which enables the analysis of haemodynamics^7^, or to determine myocardial strain rate^8^. Similarly, motion atlases, comprising spatiotemporal information, have been applied to effectively detect dilated cardiomyopathy^9^, as well as to predict responses to cardiac resynchronisation therapy^10,11^.

Distinct imaging modalities (such as CMR and 3DE) produce systematic measurement biases which can be regionally variable and further influenced by a particular analysis tool or observer^12^, due to differences in the final image appearance as a result of modality-specific acquisition and image formation processes. Therefore, to enable comparisons across datasets with varying protocols, a posteriori corrections are often applied to account for method-specific biases. One approach is to directly account for bias in the specific measurement of interest, such as the use of linear regression to determine a correction factor between outputs from the analysis of a common dataset^13^. Disadvantages of this approach are that bias correction may need to be performed several times (i.e., independently for each measurement of interest), and that it is unable to correct for biases in regional geometry. Other successful bias correction methods have been applied at the image intensity level, typically using clustering techniques^14^, or shape prior level sets to account for intensity heterogeneities in CMR images^15^. However, low-level image corrections cannot easily be applied to images obtained using different modalities, nor used to account for observer- or method-specific biases.

Extending upon previous work on correction of regional shape bias between different CMR acquisition protocols^16^, we sought to address previously quantified differences between geometries of the left ventricle (LV) derived from 3DE and CMR^12^. Using an existing database of paired multimodal cardiac images, we apply a method for spatiotemporal mapping between time-varying LV geometries over a full cardiac cycle using dynamic time warping (DTW) and partial least squares (PLS) regression, to transform geometric models derived from 3DE to best match those from CMR as the clinical gold-standard for chamber quantification. This generalised framework corrects for bias in clinical cardiac indices that can be derived from geometries reconstructed using different protocols, and thus enables study-specific cohort datasets to be indexed against larger population databases, such as the Multi-Ethnic Study of Atherosclerosis^17^ and UK Biobank^18^, regardless of bias arising from different observers, analysis tools, or imaging modalities.

## Methods

### Data acquisition

Non-invasive 3DE and CMR scans were performed consecutively within two hours in 138 participants (84 healthy controls and 54 patients with acquired, non-ischaemic cardiac disease). Ethical approval for this study was granted by the Health and Disability Ethics Committee of New Zealand (17/CEN/226), and all research was performed in accordance with relevant guidelines and regulations. Written informed consent was obtained from each participant.

Multi-planar cine CMR imaging was performed on a Siemens Magnetom 1.5 T Avanto Fit (n = 80) or 3 T Skyra (n = 58) scanner (Siemens Healthcare, Erlangen, Germany), using a balanced steady-state free precession sequence with retrospective gating. Three long-axis slices (standard two-, three-, and four-chamber views) and a short-axis stack of 6–10 slices (spanning the length of the LV from mitral valve to apex) were acquired under breath-holds. The following imaging parameters were typical: TR = 3.7 ms, TE = 1.6 ms, flip angle = 45°, field of view (FOV) = 360 mm × 360 mm, in-plane resolution = 1.4 mm × 1.4 mm, and slice thickness = 6 mm. Using these settings, an average of 29 (range 20–44) image frames were obtained per cardiac cycle across the study population.

Transthoracic 3DE image volumes were acquired using a Siemens ACUSON SC2000 Ultrasound System with a 4Z1c transducer (Siemens Medical Solutions, Mountain View, CA, USA) from the apical window in a steep left lateral decubitus position. Imaging parameters (such as FOV, focal depth, gain, compression, and frequency) were optimised for each subject to obtain real-time (single cycle) targeted LV acquisitions during breath-holds, to maximise the sampling rate while maintaining adequate spatial resolution for analysis. This produced an average of 38 (range 15–70) image frames per cardiac cycle. The duration of each acquisition was automatically determined by the R–R interval from the corresponding electrocardiogram.

### Image analysis and geometric modelling

Time-varying geometric models of the LV over one cardiac cycle were constructed semi-automatically from CMR by guide-point modelling^19^ using *Cardiac Image Modeller* (CIM, v8.1, University of Auckland, New Zealand). Briefly, analysis involved identifying anatomically homologous landmarks (i.e., base of the myocardium in the long-axis slices; apical centroid, basal centroid, and right ventricular insertion points along the LV epicardial border in the short-axis slices, where applicable), correcting in-plane breath-hold mis-registrations, and interactively fitting contours to the endocardial and epicardial borders on long- and short-axis slices. The output from this analysis consists of a previously described geometric model of the LV consisting of 16 bicubic Hermite and linear elements^20^, from which 145 unique surface points were sampled per surface (for the endocardium and epicardium) to produce 290 3D rectangular Cartesian coordinates $(\mathrm{x},\mathrm{y},\mathrm{z})$ representing the LV myocardium. All CMR analyses were conducted by a single experienced analyst.

Corresponding geometric models from 3DE were generated using a fully-automatic B-spline Explicit Active Surfaces (BEAS) algorithm^21^, whereby an initial LV shape model is evolved towards a detected edge described by a low-level energy criterion based upon the expected intensities of the blood pool and the endocardium. To obtain a motion-coherent segmentation over the full cycle, a localised anatomical affine optical flow algorithm is applied to track the model between sequential frames, which is subsequently refined through recursive block matching^22^.

Owing to variability in LV position within the image volume, all 3DE LV geometries were registered with the geometric descriptions obtained from the CMR analyses—such that the LV long-axis is oriented along the $x$-axis, with the $y$-axis directed toward the centre of the right ventricle (RV), and the origin positioned at one-third of the distance from base to apex at end-diastole (ED). To achieve this, surfaces of the geometric model were fitted to the BEAS endocardial and epicardial points using least squares minimisation, after having established the $x$ and $y$ coordinate axes. While the $x$-axis could be directly computed as a vector between the apex and basal centroids, the $y$-axis was approximated as being 70° from the inferior RV insertion (automatically detected from the images^23^), as the anterior RV wall is generally not well visualised in 3DE. This enabled a standardised description of LV geometry (i.e., 290 3D points within a common cardiac coordinate system) to be obtained from both CMR and 3DE.

### Temporal normalisation and registration

Differences in temporal resolution between CMR and 3DE, as well as within the same modality (due to subject-specific imaging parameter optimisations) resulted in a variable number of frames per acquisition. Besides the variability in sampling rate, changes in heart rate (and hence R–R interval) for the same subject between modalities were also observed. As CMR and 3DE imaging were not simultaneous, subjects naturally exhibited physiological variation between acquisitions, governed by complex mechanisms that modulate the electromechanical coupling and cardiac function^24^. Furthermore, different phases of the cardiac cycle, particularly those during diastole, do not scale uniformly with changes in the R–R interval^25^, resulting in chamber volume fluctuations that are also subject-specific.

Previous works have addressed this non-uniformity by means of piecewise linear temporal scaling that decomposes the cardiac cycle into sub-phases, which are aligned separately^7,26^. Rather than imposing these assumptions, within-subject temporal alignment was performed here by extending upon dynamic time warping (DTW)^27,28^ to minimise the discrepancy between volume traces derived from CMR and 3DE on a per-subject basis. A similar strategy has previously been applied to align cardiac sequences using an image-based surrogate for cardiac motion in the absence of chamber volume^29–31^.

To perform DTW, LV volumes over one cardiac cycle derived from the same subject are represented as two independent time series, each linearly interpolated to $n$ and $m$ uniformly spaced samples for 3DE and CMR, respectively, and normalised between 0 and 1 in magnitude according to the minimum and maximum volume:

1 $$T={t}_{1}, {t}_{2}, \dots , {t}_{i}, \dots , {t}_{n}$$

2 $$S={s}_{1}, {s}_{2}, \dots , {s}_{j}, \dots , {s}_{m}$$

where $T$ is the template signal consisting of uniformly sampled 3DE volume measurements of length $n=30$ (typical of the number of image frames per cycle obtained in cardiac imaging), to which $S$, a secondary signal of uniformly sampled CMR volume measurements of length $m=60$ (such that $S$ exhibits twice the sampling rate of $T$), is temporally warped to match, while $i$ and $j$ represent sample indices in the time domain. A $n$-by-$m$ discrepancy matrix is then formed, where the value of a given element, $d\left(i, j\right)$, is:

3 $$d\left(i, j\right){=\left({t}_{i}-{s}_{j}\right)}^{2}$$

The following dynamic programming problem is used to construct a cost matrix, where each element, $\gamma \left(i,j\right)$, is calculated using the minimum cumulative squared distance based on prior neighbouring elements:

4 $$\gamma \left(i,j\right)=d\left(i, j\right)+min\left[\gamma \left(i-1, j\right), \gamma \left(i-1, j-1\right), \gamma \left(i, j-1\right) + p\right]$$

To limit time expansions of the secondary signal (i.e., a step forward in $i$ but not $j$ despite the higher sampling rate of $S$), we introduce a penalty term, $p$, equal to the maximum difference at any given time step between normalised volume measurements in $T$ and $S$. This yields an optimal warping path, $W$, such that the value of the last element, $\gamma \left(n,m\right)$, is minimised:

5 $$W={w}_{1}, {w}_{2}, \ldots , {w}_{k}, \ldots , {w}_{o}$$

where each ${w}_{k}$ corresponds to a pair of indices $\left({i}_{k}, {j}_{k}\right)$ such that $W$ is monotonic and continuous, with fixed endpoints (i.e., ${w}_{1}=(1, 1)$ and ${w}_{o}=(n, m)$). Using $W$, it is then possible to determine a vector, ${J}^{\prime}$, containing $n$ time warped indices of $S$:

6 $${j}_{i}^{^{\prime}}=\frac{1}{\sum_{k=1}^{o}\delta ({i}_{k}, i)}\left(\sum_{k=1}^{o}\delta ({i}_{k}, i)\cdot {j}_{k} \right), \delta \left({i}_{k}, i\right)=\left\{\begin{array}{ll}0,&\quad {i}_{k}\ne i\\ 1,&\quad {i}_{k}=i\end{array}\right.$$

**Figure 1:**
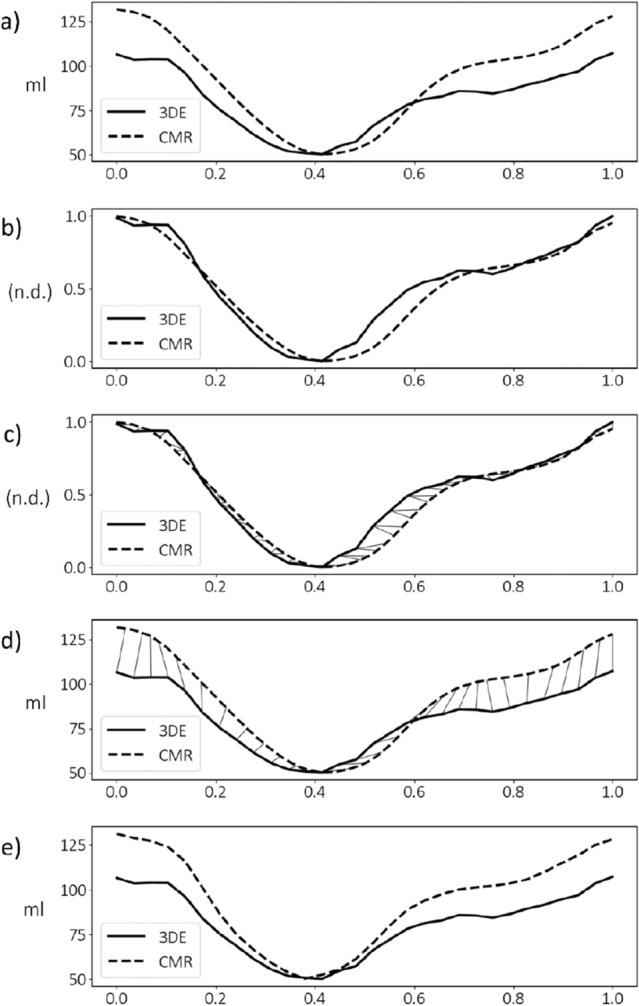
Dynamic time warping method for temporal alignment of left ventricular geometric models using volume traces over one cardiac cycle (horizontal axis) derived from 3D echocardiography (3DE) and cardiac magnetic resonance (CMR) imaging. (**a**) Volume traces from image analysis after temporal normalisation. (**b**) Volume normalisation producing traces with no dimensions (n.d.). (**c**) Warping paths between normalised volumes traces. (**d**) One-to-one mapping between volume traces. (**e**) Aligned volume traces.

Each warped timepoint, ${j}_{i}^{^{\prime}}$, is thus the mean of all ${j}_{k}$ for which ${i}_{k}=i$, yielding a one-to-one mapping between 3DE and CMR volumes. Finally, non-uniformly spaced samples of CMR geometries could be evaluated at each ${j}_{i}^{^{\prime}}$, using linear interpolation, corresponding to each of the 30 uniformly spaced 3DE geometries (also obtained by linear interpolation). An illustration of DTW is shown in Fig. 1. No between-subject temporal alignment was performed.

### Geometric mapping

Following temporal registration, PLS regression was used to calculate a geometric mapping between corresponding 3DE- and CMR-derived dynamic LV models. Having been widely adopted in chemometrics and related fields^32–34^, PLS theory has been comprehensively described in previous literature^35,36^, with a number of applications in cardiac modelling and analysis^37–40^.

Briefly, PLS regression comprises an initial dimension reduction step, followed by regression in this latent space to yield an optimal mapping between feature ($\mathbf{X}$) and target ($\mathbf{Y}$) variables. The initial decomposition problem can be presented as:

7 $$\mathbf{X = TP^\intercal}$$

8 $$ \mathbf{Y = UQ^\intercal}$$

For the present application, $\mathbf{X}$ and $\mathbf{Y}$ are matrices of time-varying LV geometry data (which are mean-centred and *z-*normalised) derived from 3DE and CMR, respectively, and each matrix consists of 138 observations (rows), and 26,100 (290 surface points × 3 spatial dimensions × 30 time frames) variables (columns). Accordingly, $\mathbf{T}$ and $\mathbf{U}$ contain the eigenvalues corresponding to the orthogonal eigenvectors in $\mathbf{P}$ and $\mathbf{Q}$, for $\mathbf{X}$ and $\mathbf{Y}$, respectively. Here, $\mathbf{X}$ and $\mathbf{Y}$ are simultaneously decomposed in an iterative process to maximise the covariance of features and targets (i.e., such that the scores in $\mathbf{T}$ exhibit the highest correlation with the corresponding scores in $\mathbf{U}$). Linear regression is then performed between $\mathbf{T}$ and $\mathbf{U}$ to yield a matrix of coefficients, $\beta$:

9 $$ \mathbf{U=T\beta}$$

from which estimates of CMR geometries, $\mathbf{\hat{Y}}$, can be computed as a function of measured 3DE geometries, $\mathbf{X}$:

10 $$\mathbf{\hat{Y} = T \beta Q^\intercal = X P \beta Q^\intercal}$$

In this work, the PLS regression step was implemented using the *scikit-learn* library^41^ with the nonlinear iterative partial least squares (NIPALS) algorithm^42^ in Python. The optimal number of PLS components was determined using five-fold cross-validation on the entire dataset, based on the smallest mean squared error between variables in $\mathbf{\hat{Y}}$ and $\mathbf{Y}$.

### Validation and mapping performance

Results were evaluated using leave-one-out cross-validation to provide an approximately unbiased estimate of mapping performance across the entire study population. Average root mean squared errors (RMSE) were computed between each set of 3D surface coordinates over all time frames to measure the similarity between spatiotemporally aligned 3DE and CMR geometries. To assess the similarity of regional LV geometry, average RMSE values were also calculated for discrete segments defined by the American Heart Association (AHA) 17-segment model^43^.

The utility of the mapping function for bias correction was also assessed by comparing routine clinical LV indices including end-diastolic volume (EDV), end-systolic volume (ESV), LV mass (LVM), ejection fraction (EF), and peak systolic global longitudinal strain (GLS) (calculated using endocardial arc lengths^44^) derived from 3DE and CMR, before and after spatiotemporal mapping was applied. Furthermore, indices calculated from the rates of change of volume and GLS (including peak ejection rate (PER), peak early filling rate (PFR_E_), peak active filling rate (PFR_A_), and peak systolic strain rate (PSR)) were used to assess the accuracy of time-dependent indices. All CMR measurements were calculated from the unaligned image-derived 3D geometric models.

Demographic variables between control and disease groups were compared using a two-sample independent *t*-test for continuous variables, and the χ^2^ test for sex. Paired-sample *t*-tests were used to identify statistically significant differences between the means of cardiac indices derived from each imaging modality, complemented with Bland–Altman analyses to illustrate the agreement between paired variables. All statistical tests were deemed significant if the two-tailed *p-*value was < 0.01. To determine the reliability of 3DE with respect to CMR before and after mapping, an intraclass correlation coefficient (ICC) based on a two-way, mixed effects model for absolute agreement^45^, was calculated for each index.

## Results

### Participant demographics

Demographics (including age, sex, and body surface area) and changes in heart rate between 3DE and CMR acquisitions are presented in Table 1. To represent a diverse range of LV geometries, the dataset included 84 healthy subjects and 54 participants with a variety of cardiac diseases (i.e., 15 patients with LV hypertrophy, 11 patients with cardiac amyloidosis, 10 patients with aortic regurgitation, 8 patients with hypertrophic cardiomyopathy, 6 patients with dilated cardiomyopathy, and 4 heart transplant recipients).

**Table 1:** Participant demographics (mean ± standard deviations, and [ranges]) for control and disease groups including age, sex, body surface area (BSA), and body mass index (BMI). The change in heart rate (HR) between CMR and 3DE acquisitions (calculated as HR_3DE_ − HR_CMR_) is provided as an indication of HR variability across the study population.

Total N = 138	Control (n = 84)	Disease (n = 54)	*P* value
Age (years)	37 ± 16 [18–74]	61 ± 15 [18–84]	< 0.001*
Male sex (frequency (%))	45 (54%)	39 (72%)	0.028
†BSA (m^2^)	1.84 ± 0.23 [1.39–2.55]	2.02 ± 0.24 [1.46–2.72]	< 0.001*
BMI (kg/m^2^)	24.4 ± 4.2 [16.9–44.4]	28.7 ± 5.5 [16.7–48.9]	< 0.001*
HR change (bpm)	− 1 ± 6 [− 18 to 15]	− 1 ± 6 [− 13 to 11]	0.608

Asterisks (*) denote statistically significant differences (*p* < 0.01).

^†^Calculated using the Mosteller formula^46^.

### Correction of global and regional geometry

**Figure 2:**
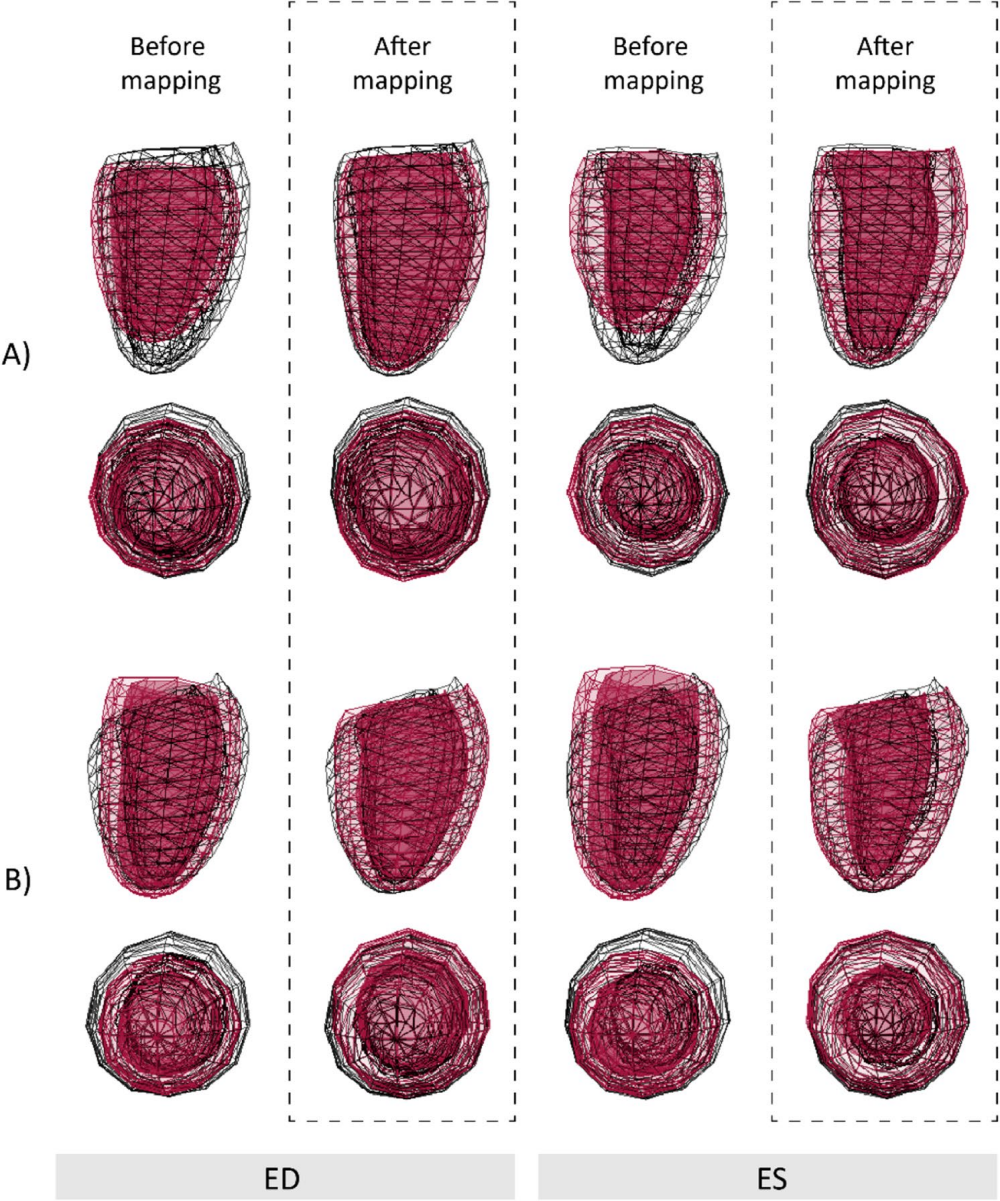
Long- and short-axis mesh overlays of left ventricular geometries derived from 3D echocardiography (red) and cardiac magnetic resonance imaging (black wireframe) acquired from two subjects before and after spatiotemporal mapping. Comparisons are shown at end-diastole (ED) and end-systole (ES). (**A**) Healthy control (39-year-old female). (**B**) Patient with transthyretin amyloidosis (84-year-old male).

Average population RMSE between surface coordinates of 3DE and CMR over the cardiac cycle decreased from 7 ± 1 mm to 4 ± 1 mm with respect to global geometry after spatiotemporal mapping, and from 6 ± 1 mm to 4 ± 1 mm, and 8 ± 1 mm to 4 ± 1 mm, for control and disease groups, respectively. Figure 2 illustrates original and mapped 3DE-derived ED and end-systole (ES) geometries for two subjects, demonstrating the greater similarity of the mapped models compared with the corresponding CMR geometries, including the effect of lengthening the foreshortened 3DE models (Fig. 2A), and adjusting for base-plane angle differences observed in certain pathologies captured by CMR (Fig. 2B). Animations for these two subjects, showing the effect of spatiotemporal mapping on the endocardial and epicardial surface points over the full cardiac cycle are shown in the Supplementary Material (Videos 1–4).

**Figure 3:**
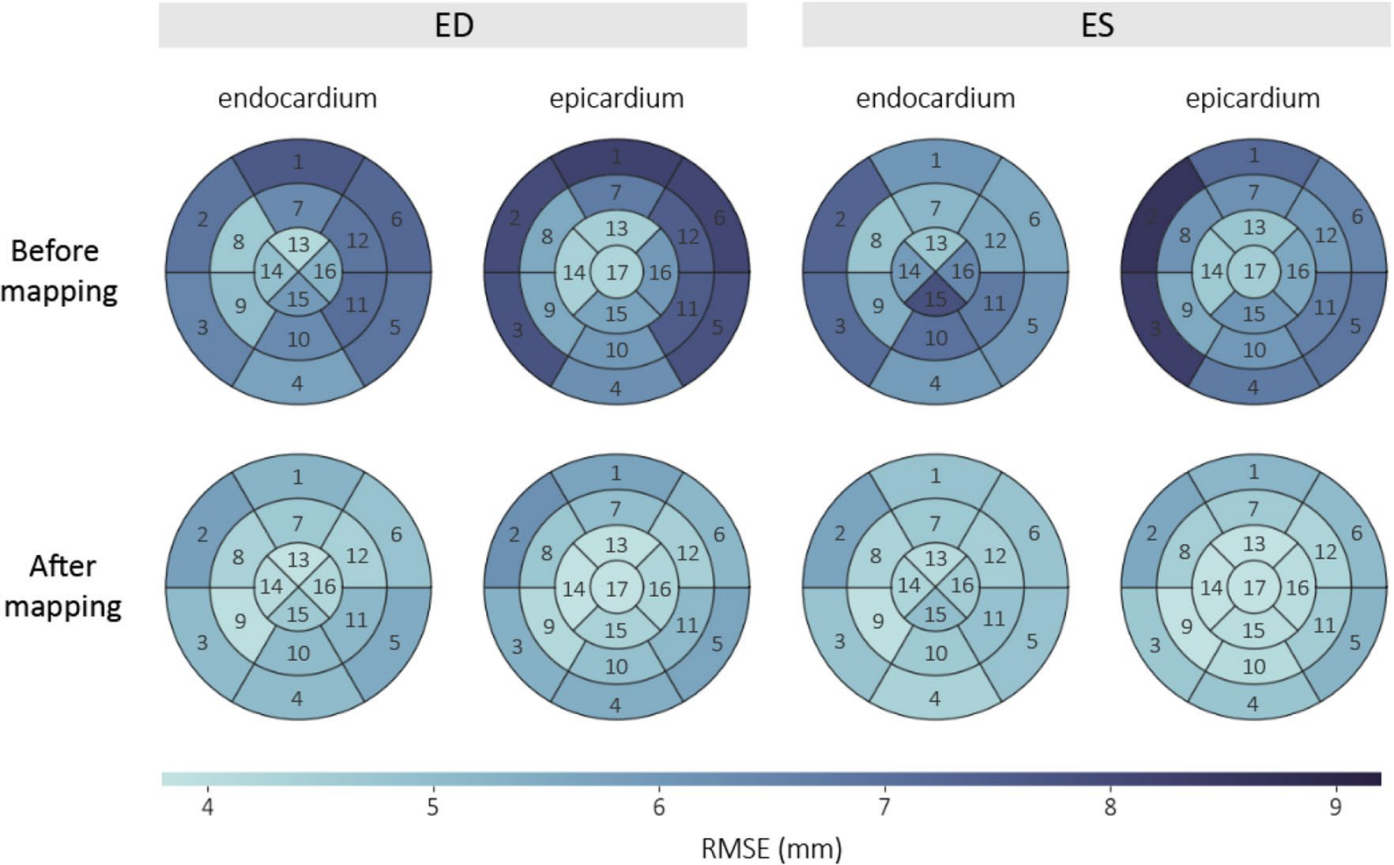
Population regional root mean squared error (RMSE) in mm between endocardial and epicardial surfaces derived from 3D echocardiography and cardiac magnetic resonance imaging at end-diastole (ED) and end-systole (ES), before and after spatiotemporal mapping. Numbers denote segments of the American Heart Association (AHA) 17-segment model^43^.

Surface distances between 3DE and CMR were quantified on a regional basis (Fig. 3), using 16 endocardial segments (excluding the apical cap) and 17 epicardial segments, as defined by the AHA model of the LV. Before mapping, the RMSE values between corresponding surfaces tended to be greater towards the basal and anterolateral aspects of the LV, whereas after mapping, the RMSE magnitudes were more homogeneously distributed across the endocardial and epicardial surfaces.

### Agreement in cardiac indices

A comparison of LV cardiac indices derived from CMR and 3DE is presented in Table 2, where each 3DE measurement was re-evaluated after spatiotemporal mapping with leave-one-out cross validation. Paired sample *t*-tests revealed statistically significant differences in the means of EDV, ESV, LVM, EF, and GLS (*p* < 0.001), between CMR and unmapped 3DE geometries produced by the BEAS algorithm. Spatiotemporal mapping resulted in substantially smaller biases, such that no statistically significant differences were observed between CMR and mapped 3DE geometries. In all instances, this was accompanied by higher ICC values.

**Table 2:** Population cardiac magnetic resonance (CMR) imaging summary and relative 3D echocardiography (3DE) biases (mean ± standard deviation), with intraclass correlation coefficients (ICC) for left ventricular end-diastolic volume (EDV), end-systolic volume (ESV), mass (LVM), ejection fraction (EF), and global longitudinal strain (GLS), before and after spatiotemporal mapping. Indexed values are also provided in squared brackets, where applicable.

N = 138	EDV (ml [ml/m^2^])	ESV (ml [ml/m^2^])	LVM (g [g/m^2^])	EF (%)	GLS (%)
CMR	149 ± 38 [77 ± 15]	59 ± 23 [31 ± 11]	135 ± 48 [69 ± 20]	61 ± 8	− 19 ± 4
3DE bias (before)	*− 16 ± 20 [*− 9 ± 10]	*13 ± 19 [*6 ± 9]	*− 11 ± 32 [*− 6 ± 17]	*− 14 ± 8	*5 ± 4
3DE bias (after)	− 4 ± 19 [− 2 ± 10]	0 ± 15 [0 ± 7]	− 1 ± 23 [2 ± 12]	− 1 ± 6	0 ± 3
ICC (before)	0.890 [0.803]	0.802 [0.742]	0.839 [0.732]	0.355	0.458
ICC (after)	0.915 [0.855]	0.856 [0.809]	0.931 [0.878]	0.645	0.658

Asterisks (*) denote statistically significant differences compared to CMR-derived indices (*p* < 0.01).

To investigate mapping performance with respect to pathological status, the above statistical analyses were repeated for control (Table 3) and disease (Table 4) sub-populations independently. As with the mapping performance on the total population in Table 2, lower magnitudes of 3DE bias were observed across all volumetric and functional indices after mapping was applied, aside from LVM in the control group which exhibited a small increase in the magnitude bias from − 1 to 4 g (although this did not produce a statistically significant difference with respect to CMR). Similarly, higher ICC values were generally observed across all cardiac indices for both groups, with the exception of EDV (but not indexed EDV) in the disease group.

**Table 3:** Control group cardiac magnetic resonance (CMR) imaging summary and relative 3D echocardiography (3DE) biases (mean ± standard deviation), with intraclass correlation coefficients (ICC) for left ventricular end-diastolic volume (EDV), end-systolic volume (ESV), mass (LVM), ejection fraction (EF), and global longitudinal strain (GLS), before and after spatiotemporal mapping. Indexed values are also provided in squared brackets, where applicable.

n = 84	EDV (ml [ml/m^2^])	ESV (ml [ml/m^2^])	LVM (g [g/m^2^])	EF (%)	GLS (%)
CMR	141 ± 33 [76 ± 13]	54 ± 16 [29 ± 7]	113 ± 31 [61 ± 12]	62 ± 5	− 21 ± 3
3DE bias (before)	*− 19 ± 17 [*− 11 ± 9]	*9 ± 14 [*5 ± 8]	− 1 ± 22 [− 1 ± 12]	*− 13 ± 7	*5 ± 4
3DE bias (after)	*− 5 ± 17 [− 3 ± 9]	− 1 ± 10 [0 ± 6]	4 ± 18 [3 ± 10]	− 1 ± 4	1 ± 3
ICC (before)	0.850 [0.722]	0.797 [0.675]	0.867 [0.744]	0.278	0.301
ICC (after)	0.911 [0.827]	0.871 [0.782]	0.907 [0.783]	0.621	0.520

Asterisks (*) denote statistically significant differences compared to CMR-derived indices (*p* < 0.01).

**Table 4:** Disease group cardiac magnetic resonance (CMR) imaging summary and relative 3D echocardiography (3DE) biases (mean ± standard deviation), with intraclass correlation coefficients (ICC) for left ventricular end-diastolic volume (EDV), end-systolic volume (ESV), mass (LVM), ejection fraction (EF), and global longitudinal strain (GLS), before and after spatiotemporal mapping. Indexed values are also provided in squared brackets, where applicable.

n = 54	EDV (ml [ml/m^2^])	ESV (ml [ml/m^2^])	LVM (g [g/m^2^])	EF (%)	GLS (%)
CMR	160 ± 41 [79 ± 18]	68 ± 29 [34 ± 14]	168 ± 50 [83 ± 23]	59 ± 10	− 17 ± 4
3DE bias (before)	*− 11 ± 22 [− 6 ± 11]	*19 ± 23 [9 ± 11]	*− 26 ± 39 [− 13 ± 20]	*− 16 ± 10	*5 ± 5
3DE bias (after)	− 3 ± 23 [− 1 ± 11]	0 ± 20 [0 ± 10]	− 9 ± 28 [− 5 ± 14]	− 1 ± 8	− 1 ± 4
ICC (before)	0.911 [0.873]	0.765 [0.741]	0.754 [0.642]	0.355	0.310
ICC (after)	0.905 [0.875]	0.815 [0.802]	0.906 [0.862]	0.590	0.565

Asterisks (*) denote statistically significant differences compared to CMR-derived indices (*p* < 0.01).

**Figure 4:**
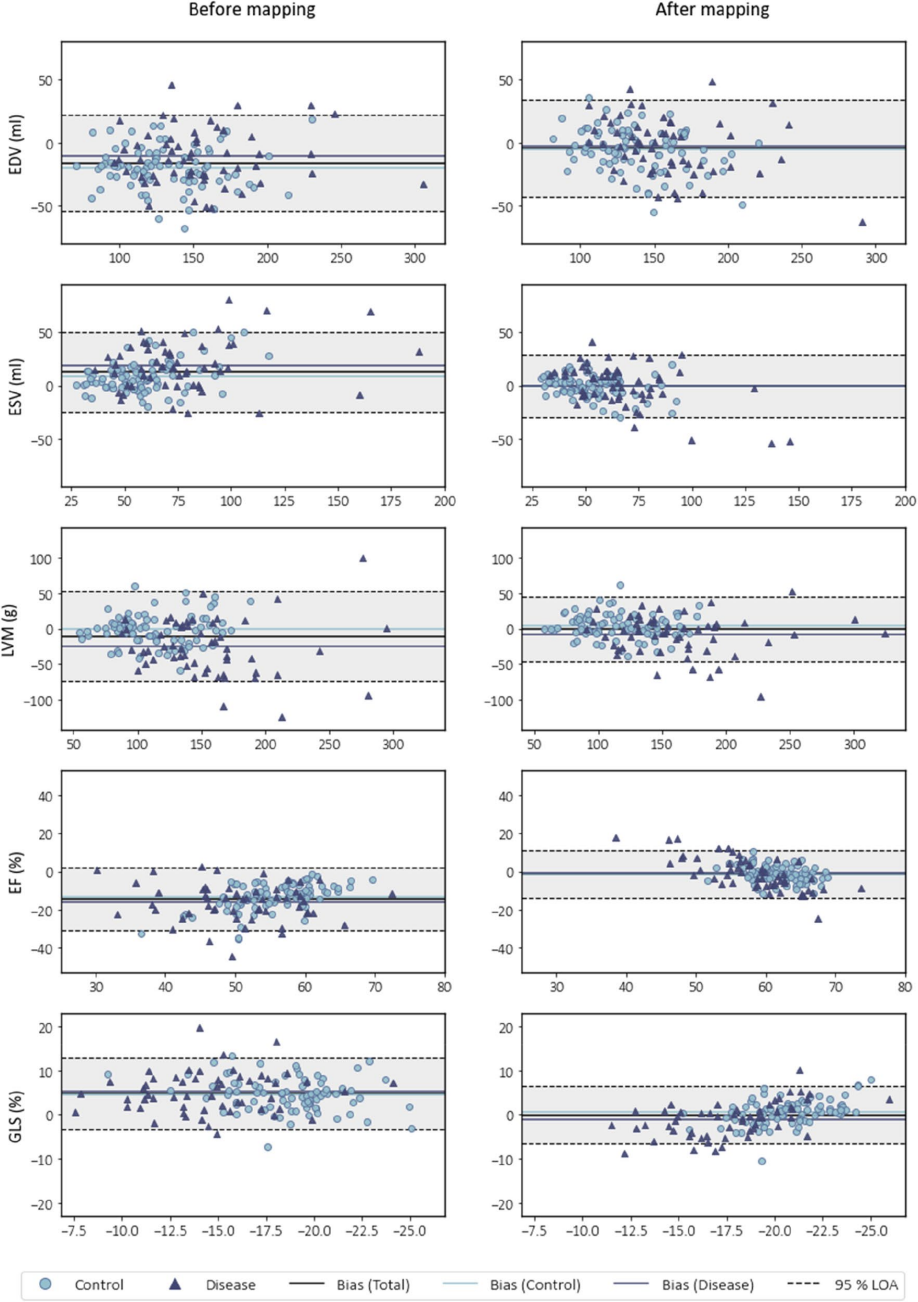
Bland–Altman plots showing biases and 95% limits of agreement (LOA), with vertical axes representing differences between 3D echocardiography (3DE) and cardiac magnetic resonance (CMR) imaging, plotted against horizontal axes representing the mean of measures obtained from 3DE and CMR. Left and right columns show comparisons before and after spatiotemporal mapping of 3DE geometries for left ventricular end-diastolic volume (EDV), end-systolic volume (ESV), mass (LVM), ejection fraction (EF), and global longitudinal strain (GLS).

Bland–Altman plots with measurement samples stratified by group are shown in Fig. 4. For the mapped 3DE geometries, the analyses revealed lower mean bias compared to the unmapped geometries, and narrower 95% limits of agreement for all cardiac indices with respect to the total study population.

### Volume and strain traces

**Figure 5:**
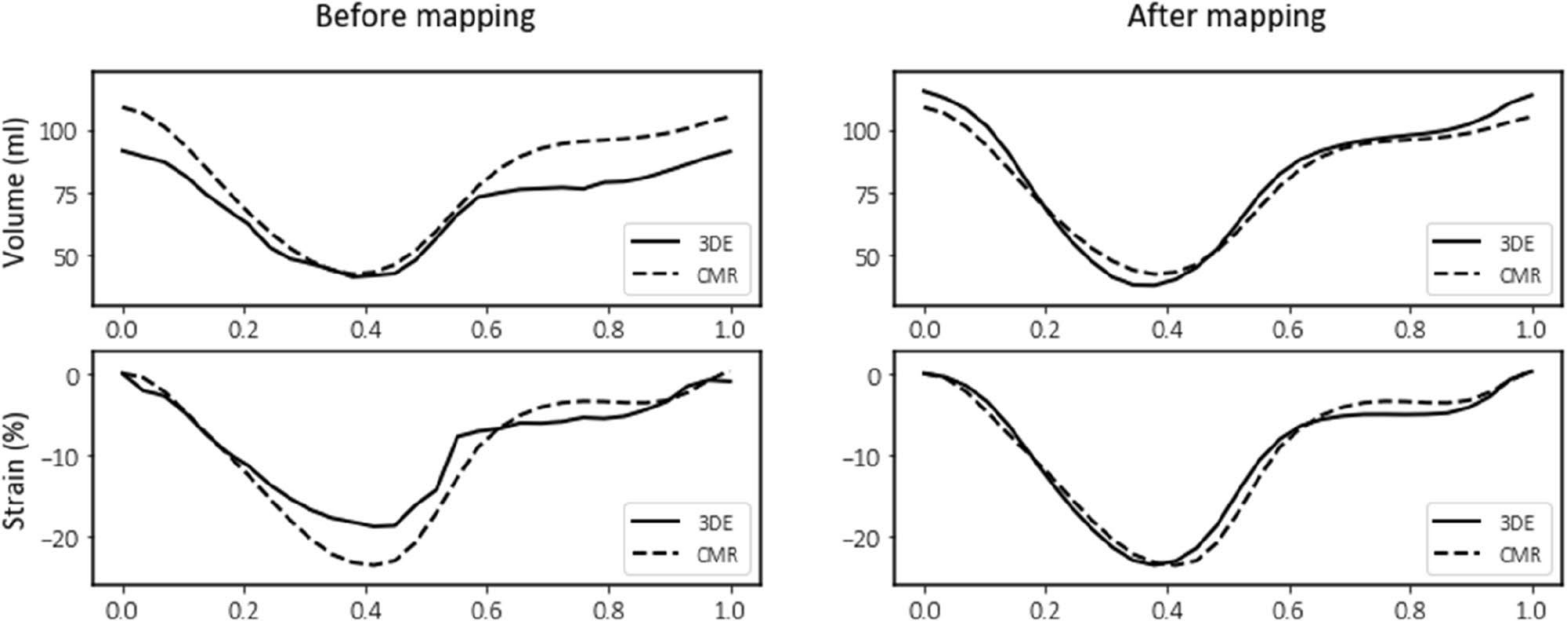
Comparison of volume and strain traces derived from cardiac magnetic resonance (CMR) imaging (dashed lines) and 3D echocardiography (3DE) (solid lines) before and after spatiotemporal mapping over one cardiac cycle from a healthy control subject (38-year-old female).

The volume and strain traces derived from the mapped 3DE geometries exhibited a greater degree of similarity to those obtained using CMR than those derived from the original 3DE geometries, with an example shown in Fig. 5. The degree of similarity between 3DE and CMR was quantified using the cumulative DTW distance over one cardiac cycle (consisting of 30 uniform samples), which decreased from 360 ± 244 to 224 ± 192 ml for volume, and 55 ± 38% to 29 ± 15% for strain (for the total population), after mapping was applied.

A comparison of indices derived from these traces (i.e., PER, PFR_E_, PFR_A_, and PSR) calculated from 3DE geometries before and after mapping, to those from corresponding CMR acquisitions, are presented in Table 5. Smaller magnitudes of bias after mapping were observed for PER and PSR, but not for PFR_E_ (where there was a larger magnitude of bias) and PFR_A_ (where the direction of bias was reversed, but the magnitude of bias was similar). However, mapping resulted in all indices exhibiting smaller standard deviations of differences (interpreted as narrower limits of agreement) between CMR and 3DE, as well as higher ICC values, suggesting the improved reliability of measurements after spatiotemporal mapping of geometries was applied.

**Table 5:** Population cardiac magnetic resonance (CMR) summary and relative 3D echocardiography (3DE) biases (mean ± standard deviation), with intraclass correlation coefficients (ICC) for left ventricular peak ejection rate (PER), peak early filling rate (PFR_E_), peak active filling rate (PFR_A_), and peak systolic strain rate (PSR), before and after spatiotemporal mapping.

N = 138	PER (ml s^−1^)	PFR_E_ (ml s^−1^)	PFR_A_ (g s^−1^)	PSR (s^−1^)
CMR	− 409 ± 108	341 ± 103	181 ± 84	− 0.87 ± 0.18
3DE bias (before)	*60 ± 93	2 ± 128	20 ± 103	*0.13 ± 0.22
3DE bias (after)	12 ± 86	*− 38 ± 89	*− 20 ± 68	0.01 ± 0.19
ICC (before)	0.724	0.641	0.532	0.467
ICC (after)	0.775	0.651	0.684	0.504

Asterisks (*) denote statistically significant differences (*p* < 0.01).

## Discussion

Spatiotemporal mapping of LV geometries using DTW and PLS regression is an effective method for bias correction of cardiac indices derived from different cardiac imaging modalities. By mapping cardiac geometry directly, this approach corrects for potential biases in geometry-derived measurements (such as functional cardiac indices), which do not have to be specified a priori. As protocol-dependent biases can be both global and regional in nature (see Fig. 3), acting on local geometric information enables region-specific bias correction for applications such as the comparison and quantification of regional wall motion, or to capture subtle morphological changes in LV shape.

The use of DTW for the temporal alignment of cardiac sequences confers several advantages. Firstly, DTW is automated, does not require a temporal cardiac template, and is consequently robust to cases of abnormal systolic or diastolic function, which may produce atypical volume traces^47,48^. Similarly, relationships between cardiac output and mechanisms that regulate stroke volume and heart rate are, in general, non-trivial, and difficult to predict per subject. For instances in which recordings are non-synchronous, heart rate variability is expected, particularly in healthy subjects^49^. Furthermore, the scaling of the various segments of the cardiac cycle with respect to R–R interval exhibits high inter-subject variability and subsequently has not been universally modelled with success^50,51^. By performing temporal warping on a subject-specific basis, this method does not rely on assumptions regarding changes in the volume curve arising from beat-to-beat variation. An alternative subject-specific approach to address the challenge associated with non-synchronous cardiac cycles is to exploit homologous temporal landmarks based on distinct mechanical and electrical events^52,53^. While this method ensures physiological constraints, it relies on the correct identification of timings associated with valve opening and closing, which can be hindered by poor image quality. Likewise, the detection of certain electrocardiogram features (e.g., P-wave) is not always feasible in the presence of rhythm irregularities such as atrial fibrillation. While in this work temporal registration was achieved by aligning LV cavity volume, DTW in theory can be applied to any time-varying geometries exhibiting cyclic behaviour (and from which a cyclical signal can be derived if temporal alignment is required).

Using paired CMR and 3DE data from 138 unique subjects, we demonstrate that PLS regression can effectively correct for biases in cardiac indices, despite these measurements not being explicitly included in the generation of the mapping function. Comparisons of routine cardiac indices from both healthy controls and a heterogeneous disease group showed a significant reduction in mean bias, narrower limits of agreement, and improved measurement reliability (with only minor exceptions from this trend) after spatiotemporal mapping was applied to the 3DE-derived geometries. This approach for bias correction enables targeted studies to perform quantitative comparisons against larger databases to obtain population-based outcomes, as well as the pooling and re-use of smaller datasets. The use of PLS regression for constructing predictive models has been shown to be particularly effective when variables are highly collinear, or when the number of response variables greatly exceeds the number of observations^54^. This is the case for the present application, where features in $\mathbf{X}$ are intrinsically associated with adjacent geometric coordinates in the predefined pointset (as well as across time frames), while there are a limited number of training samples, i.e., 138 subjects, compared with 26,100 target variables in $\mathbf{Y}$. In the domain of medical image analysis, PLS regression is particularly well suited, since the challenge of limited samples is often encountered, owing to the high costs associated with data acquisition.

Once computed, the resultant mapping function can be applied to geometric datasets wherein the target imaging modality is unavailable, such as to adjust 3DE geometries in the absence of CMR. From a clinical perspective, spatiotemporal mapping may also enable reference ranges established for one modality to be applicable to another modality. For example, this approach enables the transfer of normative values derived from large population databases of CMR^18^ for application to 3DE imaging, where accepted reference values have yet to be established for some indices. This is possible without incurring costs associated with undertaking further population studies to establish modality-specific normative ranges (on the basis that a database of images acquired using the modalities of interest for the same subjects is available to derive the mapping function). Likewise, bias correction may improve measurement accuracy for serial patient follow-up performed across different imaging modalities, as well as account for changes in institutional imaging protocols.

### Future work

While we have demonstrated the correction of biases in routine cardiac indices, this method could benefit from further validation to assess the agreement for more complex analyses, such as in estimated tissue properties using biomechanics workflows, or in resultant pressure-volume loops for haemodynamic assessment, after spatiotemporal mapping is applied.

For generalisability, PLS regression is performed directly on Cartesian coordinates resulting from the image analysis of 3DE and CMR. However, other forms of geometric parameters may be more effective (and potentially more appropriate) for certain applications or anatomical structures. Further experiments using other forms of geometry, such as mapping of parameters belonging to 3D anatomical scaffolds^55^, or of alternate coordinate systems, such as prolate spheroidal coordinates commonly used to describe LV geometry^56–58^, may be beneficial. In the present example, both $\mathbf{X}$ and $\mathbf{Y}$ have the same number of variables (though this does not have to be the case for PLS regression in general), and feature selection may help to reduce variable redundancy. Where larger datasets are concerned, PLS regression may be preceded by a dimension reduction step, such as principal component analysis, in order to reduce computation time.

## Conclusions

Differences in acquisition and analysis protocols often result in measurement biases, which can manifest as statistically significant differences in clinical indices of interest, even where the analysis of an identical cohort is concerned. We present a generalised method for spatiotemporal mapping between full-cycle cardiac sequences derived from multiple imaging modalities. By mapping the cardiac geometry directly, the proposed bias correction scheme is consequently agnostic to the specific indices or anatomical region of interest, and is equally applicable regardless of pathological status.

### Supplementary Information

Supplementary Video 1.

Supplementary Video 2.

Supplementary Video 3.

Supplementary Video 4.

## Data Availability

The datasets used and/or analysed during the current study are available from the corresponding author on reasonable request.

## Abbreviations

3DE: 3D echocardiography
AHA: American Heart Association
BEAS: B-spline Explicit Active Surfaces
CMR: Cardiac magnetic resonance
DTW: Dynamic time warping
ED: End-diastole
EDV: End-diastolic volume
EF: Ejection fraction
ES: End-systole
ESV: End-systolic volume
FOV: Field of view
GLS: Global longitudinal strain
HR: Heart rate
ICC: Intraclass correlation coefficient
LV: Left ventricle
LVM: Left ventricular mass
NIPALS: Nonlinear iterative partial least squares (algorithm)
PER: Peak ejection rate
PFR_A_: Active peak filling rate
PFR_E_: Early peak filling rate
PLS: Partial least squares
PSR: Peak strain rate
RMSE: Root mean squared error
RV: Right ventricle
