# Publisher Correction: Correcting bias in cardiac geometries derived from multimodal images using spatiotemporal mapping

**DOI:** 10.1038/s41598-023-40773-7

**Published:** 2023-08-21

**Authors:** Debbie Zhao, Charlène A. Mauger, Kathleen Gilbert, Vicky Y. Wang, Gina M. Quill, Timothy M. Sutton, Boris S. Lowe, Malcolm E. Legget, Peter N. Ruygrok, Robert N. Doughty, João Pedrosa, Jan D’hooge, Alistair A. Young, Martyn P. Nash

**Affiliations:** 1 Auckland Bioengineering Institute, University of Auckland, 70 Symonds Street, Grafton, Auckland, 1010 New Zealand; 2 Department of Anatomy and Medical Imaging, University of Auckland, Auckland, New Zealand; 3 Counties Manukau Health Cardiology, Middlemore Hospital, Auckland, New Zealand; 4 Green Lane Cardiovascular Service, Auckland City Hospital, Auckland, New Zealand; 5 Department of Medicine, University of Auckland, Auckland, New Zealand; 6 Institute for Systems and Computer Engineering, Technology and Science (INESC TEC), Porto, Portugal; 7 Department of Cardiovascular Sciences, KU Leuven, Leuven, Belgium; 8 Department of Biomedical Engineering, King’s College London, London, UK; 9 Department of Engineering Science, University of Auckland, Auckland, New Zealand

Correction to: *Scientific Reports* 10.1038/s41598-023-33968-5, published online 19 May 2023

The original PDF version of this Article contained formatting errors in Equation 10.

$$\hat{\mathbf{Y}}=T\beta {Q}^{\intercal }=XP\beta {Q}^{\intercal }$$

now reads:

$$\hat{\mathbf{Y}}=\mathbf{T}\beta {\mathbf{Q}}^{\intercal }=\mathbf{X}\mathbf{P}\beta {\mathbf{Q}}^{\intercal }$$

Additionally, the Funding section in the original version of this Article was omitted. The Funding section now reads:

“This research was funded by the Health Research Council of New Zealand (programme grant 17/608) and National Heart Foundation of New Zealand (project 1834).”

The original Article has been corrected.

