# Supplementary figures and images for: Correcting bias in cardiac geometries derived from multimodal images using spatiotemporal mapping
Source: Sci Rep. 2023 May 19;13:8118. doi: 10.1038/s41598-023-33968-5 (PMC10199025; doi:10.1038/s41598-023-33968-5)

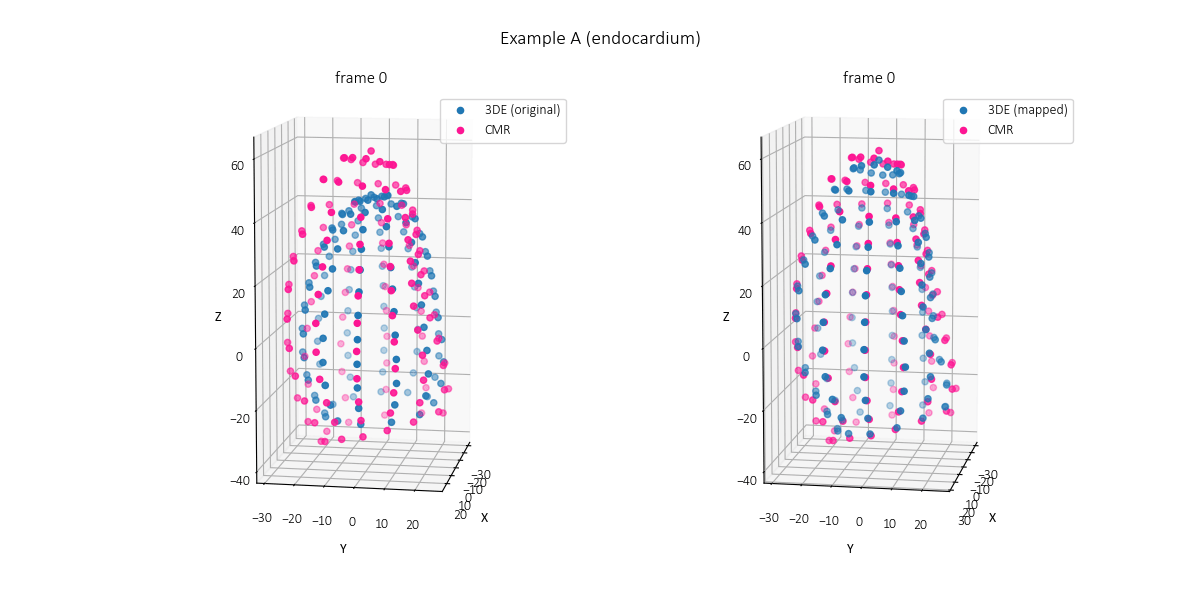

Supplement: Supplementary file 1 — Supplementary Video 1. [file 41598_2023_33968_MOESM1_ESM.gif]

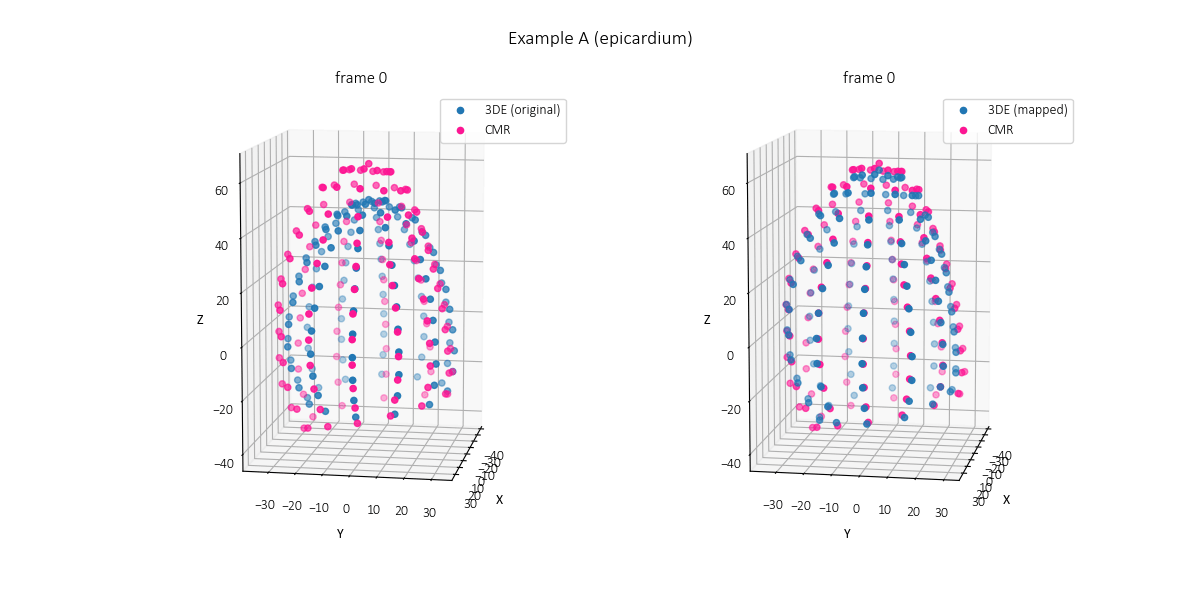

Supplement: Supplementary file 2 — Supplementary Video 2. [file 41598_2023_33968_MOESM2_ESM.gif]

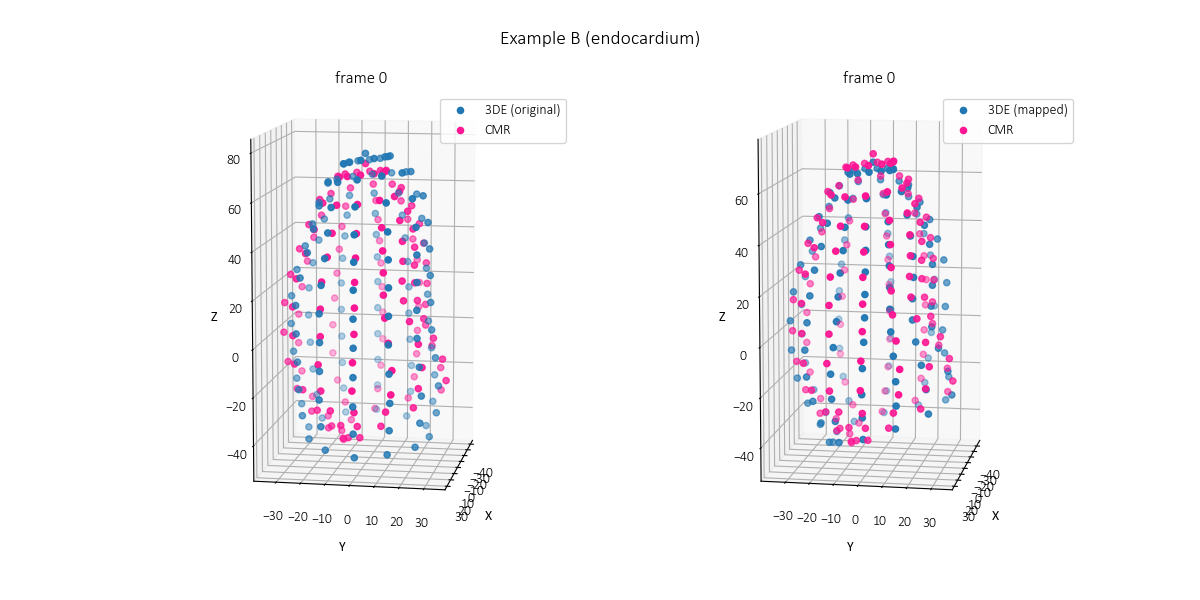

Supplement: Supplementary file 3 — Supplementary Video 3. [file 41598_2023_33968_MOESM3_ESM.gif]

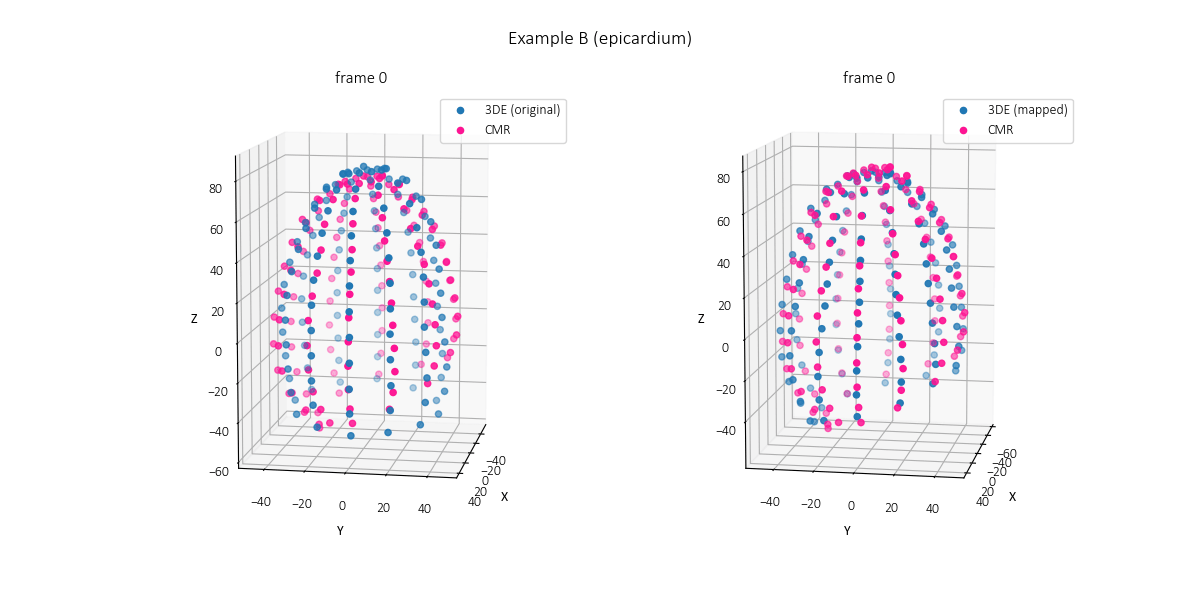

Supplement: Supplementary file 4 — Supplementary Video 4. [file 41598_2023_33968_MOESM4_ESM.gif]
